# Traumatic Brain Injury Has Not Prominent Effects on Cardiopulmonary Indices of Rat after 24 Hours: Hemodynamic, Histopathology, and Biochemical Evidence

**DOI:** 10.6091/ibj.13222.2014

**Published:** 2014-10

**Authors:** Hamid Najafipour, Ali Siahposht Khachaki, Mohammad Khaksari, Beydolah Shahouzehi, Siyavash Joukar, Hamid Reza Poursalehi

**Affiliations:** 1 *Physiology Research Center, Institute of Neuropharmacology, Kerman University of Medical Sciences, Kerman, Iran;*; 2 *Department of Physiology, Shahid Beheshti University of Medical Sciences, Tehran, Iran;*; 3 *Neuroscience Research Center, Institute of Neuropharmacology, Kerman University of Medical Sciences, Kerman, Iran*

**Keywords:** Brain injury (TBI), Cardiopulmonary, Myocardial contraction, Cytokines

## Abstract

**Background: **Accidents are the second reason for mortality and morbidity in Iran. Among them, brain injuries are the most important damage. Clarification of the effects of brain injuries on different body systems will help physicians to prioritize their treatment strategies. In this study, the effect of pure brain trauma on the cardiovascular system and lungs 24 hours post trauma was assessed. **Methods**: Male Wistar rats (n = 32) were divided into sham control and traumatic brain injury (TBI) groups. In TBI animals, under deep anesthesia, a blow to the head was induced by the fall of a 450 g weight from 2 m height. Twenty four hours later, heart electrocardiogram and functional indices, cardiac troponin I, IL-6, TNF-, IL-Iβ in tissue and serum, and the histopathology of heart and lung were assessed. **Results:** The results showed that none of the functional, biochemical, inflammatory, and histopathology indices was statistically different between the two groups at 24 hours post TBI. Indices of impulse conduction velocity in atrium (P wave duration and P-R interval) were significantly longer in the TBI group. **Conclusion:** Overall, no important functional and histopathologic disturbances were found in heart and lung of TBI group after 24 hours. If the data is reproduced in human studies, the medical team could allocate their priority to treatment of brain disorders of the victim in the first 24 hours of pure TBI and postpone extensive assessment of heart and lung health indices to later time, thus reducing patient and health system expenditures.

## INTRODUCTION

Accidents are the second reason for mortality and morbidity in Iran that cause a lot of expenditures for patients and the health system [1]. Among them, brain injuries are the most important causes of mortality and morbidity. Intense cardiovascular damages such as myocardial injuries have been reported following severe cerebrovascular disorders such as sub-arachnoid hemorrhage (SAH) [2]. Findings show that SAH causes a rise in blood pressure, heart rate, intracranial pressure, plasma norepinephrine, and cardiac troponin I (cTNI), a heart-cell injury index, accompanied by abnormal electrocardiogram (ECG) in three hours [3]. Also, brain trauma brings about significant ultra-structural changes in heart myocytes in 24 hours [4]. In another studies, it has been demonstrated that increase in cTNI after SAH is related to a rise in cardiopulmonary complication risks, delayed brain ischemia, and pulmonary congestion [5, 6]. This finding shows a close relation between heart and lung as, and the functional effects of disturbances in brain function on these two vital organs. Sakr *et al.* [7] have shown that following SAH in patients, after a sinus tachycardia, abnormal rhythms in the form of sinus bradycardia, premature ventricular contraction, and ventricular fibrillation are seen. It has been shown that in SAH patients, Q-T interval is very long, which may be a sign of cardiomyopathy [8]. In addition, increase in cTNI is related to heart systolic and diastolic functional disorders [9]. Brain ischemia may cause disorder in heart function, and this is accompanied by high levels of norepinephrine in heart and serum [10] activating beta adrenoreceptors and also by increase in cardiac and cerebral oxygen demands. The current evidence suggests the beneficial effect of beta blockers following traumatic brain injury (TBI). It has been proposed recently that parasympathetic disorders and inflammatory factors may be effective in heart myocyte injuries following SAH [11]. A more recent study also verifies that inflammation may play a role [12].

Past studies mostly have focused on the effect of SAH model of brain damage and on the heart electrical and blood biochemical factors in this model [7, 8, 11]; however, no direct heart muscle function has been assessed. In TBI brain injury models, survival rate and biochemical indices were assessed in one study [13]. Larson and colleagues [14] have recently examined the effect of TBI on systolic blood pressure (assessed by tail-cuff measurement), left ventricle ejection fraction (using ECG-gated cardiac MRI), and the production of reactive oxygen species in myocardium after TBI in rats. However in their study, the method used for TBI was fluid percussion injury through applying a liquid pressure of 49 PSI (about 2500 mmHg) over only 0.5 seconds around brain (intracranially). The measure-ments were also carried out 48 hours (for blood pressure and ejection fraction) or seven days (for reactive oxygen species) post TBI. All three variables were increased significantly during this period.

In the present study, a diffuse severe TBI model, which is more suitable than other brain injury models (such as SAH or fluid percussion injury) was chosen, as this model bears great resemblance to brain injuries caused by car and occupational accidents in humans. In addition, for the first time, direct heart function indices such as left ventricle systolic and diastolic pressures, heart muscle contraction velocity (+dp/dt max), and relaxation velocity (-dp/dt max) along with cardiac histopathology indices and cTNI, a specific heart-cell injury index, were included in the assessment of this study. Finally, to assess the relations between the above mentioned variables and the heart electrical, biochemical and inflammatory indices, ECG, cTNI levels in serum, and important inflammatory interleukins (IL-1β, IL-6, and TNF-α) in serum and heart tissue were measured. These measurements will help to identify the probable mechanisms of functional disorders in this kind of brain contusion. It has been shown that there is an inflammatory link between brain injuries and myocyte dysfunction [11, 12]. On the other hand, as lungs are among the vital organs, whose health is very important in brain trauma conditions; therefore, their histological and inflammatory indices were also assessed. Lungs have mostly not been given attention in previous brain trauma studies. Therefore, what this study is assessing is the probable disturbances in functional, electrical, histopathologic and biochemical indices of heart and also changes in histopathologic, and inflammatory indices of lungs in the first 24 hours, in which the brain edema reaches its maximum [15] and is a clinically golden time for treatment after TBI. The model used in this study is pure traumatic brain damage in rat that is very similar to pure brain injuries caused by car and occupational accidents in humans.

## MATERIALS AND METHODS

The study was carried out on 32 male Wistar rats weighing 250-300 g. The animals were randomly assigned to two main groups as follow:

1. Sham control group (n = 16): these animals were not subjected to brain injury and underwent only surgical procedure and the following indices were measured as pre-contusion indices.
2. TBI group (TBI) (n = 16): these animals were subjected to acute, diffuse brain injury and the following indices were measured as post-injury indices.

The animals of each main group were again randomly assigned into the following subgroups:

1- Assessment of Troponin I, and heart and lungs histopathology and inflammatory indices (n = 8)

2- Assessment of heart functional and electrocardiographic indices (n = 8)

All experimental procedures were carried out in accordance with the national guidelines for conducting animal studies (Ethic committee permission no 90/143KA, Kerman University of Medical Sciences, Kerman, Iran). Heart electrical and functional activity, heart troponin I, and inflammatory interleukin levels, and heart and lung histology indices were assessed. Troponin I and inflammatory cytokine levels in serum were also measured. 

***Brain injury method (TBI). ***The animals were anesthetized by adding 2-4% halothane to respiratory air. After intra-tracheal intubation through the mouth, the tracheal cannula was attached to respiratory pump for controlling breath and avoiding hypoxia. Body temperature was maintained at 37°C by placing the animals on a steel surface of surgical table with an underlying controlled heating system. The scalp was incised to reveal skull and a steel disk with thickness of 3 mm and 10-mm diameter was glued to the bone centrally along the coronal between bregma and lambda using polyacrylamide glue in order to distribute the blow onto a larger area. The animal was then laid on its stomach on a 10-mm thick foam mattress, and the blow was applied to the animal head according to the Marmarou *et al.* method [16]. In this method, a 450-g weight was dropped onto the steel disk through a tube from 2 m height. The steel disk was removed from the skull, and the animal was attached to an artificial respiration machine directly after the blow so as to avoid respiration disturbance caused by contusion in the brain respiratory centers. Our previous studies have confirmed the effectiveness of this model of TBI [17, 18]. For instance, TBI caused an increase in intracranial pressure from 5.8 ± 0.34 to 13.58 ± 0.68 mmHg and a decrease in veterinary comma scale from 15 to 9.57 ± 0.57 mmHg after four hours [17]. Also, brain tissue Evans blue content, as an index of blood-brain barrier permeability damage was increased from 27.5 ± 1 to 36.5 ± 0.5 μg/g tissue after five hours [18]. After resumption of automatic respiration, the animal was detached from the respiratory pump and placed in its own cage [19]. The following factors were measured 24 hours after blow.

***Histopathologic indices and measurement of cardiac troponin-I in serum and interleukins in tissues. ***Twenty four hours after TBI, animals were sacrificed under deep anesthesia, which was induced by sodium thiopental (50 mg/kg, i.p.). Blood sample was collected in a tube for serum separation, and the heart and lung were incised and rinsed with normal saline. The heart was halved horizontally, and one-half along with one lung was sent to pathology after being fixed in 10% formalin. The other half of the heart and one lung were immediately immersed in liquid nitrogen and preserved at -80°C for measurement of tissue interleukins until examination. Blood sample was centrifuged at 3000 ×g for 15 minutes, and serum was kept at -80°C until final analysis. Rat troponin-I was measured using ELISA method [20]. Four-micrometer-thick slices were made from tissue samples and then stained with hematoxylin-eosin. Congestion and hemorrhage, hyper eosinophilia bundle, leukocyte infiltration, and necrosis indices were assessed under four categories, including severe (+++), moderate (++), mild (+), and normal (0) [21]. One point was given to each +, and the total histological score would amount to maximum 12 points for each animal. 

***Measuring IL-6, TNF-*** ***, and IL-1β in serum and heart and lung tissues. *** For homogenization, 50 mg

 heart muscle or lung tissue was homogenized on ice in 1 ml buffer (10 mM NaCl, 20% glycerol, 20 mM HEPES, 1.5 mM MgCl_2_, 0.1 % Triton X-100, and 1 mM dithiothreitol, pH 7.4) by a homogenizer and was then centrifuged at 2500 ×g at 4°C for 1 min. The supernatant was separated and the IL-6, TNF-α, and IL-1β levels were measured in supernatant and in serum samples using appropriate kits for rats by ELISA method according to the kit instructions.

***Electrocardiogram recording and myocardial contractility indices*** ***. ***In the second subgroup, after anesthesia, ECG was recorded by attaching ECG electrodes to appropriate points. Changes in ECG, such as changes in T wave, rise or fall of ST segment, and changes in Q wave, and the wave amplitude and durations were measured by ECG analyzer software (Powerlab, Australia). Blood pressure was measured by cannulation of carotid artery and connecting the cannula to a pressure transducer and Powerlab Physiograph (ADInstruments, Australia). Mean arterial blood pressure was calculated by adding one-third of pulse pressure to the diastolic pressure [22]. In order to measure left ventricle pressure, the cannula in the carotid artery was slowly pushed forward into the left ventricle to record ventricle pressure. The Power Lab system automatically calculated the maximum ventricle contraction velocity (+dp/dt max) and maximum ventricle relaxation velocity (-dp/dt max) from ventricle pressure [21].

***Data analysis. ***SPSS 15 software was used for statistical analysis. Results were reported in mean ± SE. Independent *t*-test was used for comparing quantitative variables (e.g. blood pressure, heart contractility indices, interleukin levels, and ECG variables). Chi-square test was also used for histology indices. The level of significance was taken as *P*<0.05.

## RESULTS

***Hemodynamic variables. ***Cardiac contractility indices and arterial blood pressure of sham control and TBI groups 24 hours after TBI are shown in Table 1. No statistically significant differences were found between the two groups in either of these variables. 

**Table 1:** Hemodynamic variables (mean ± SE) in study groups (n = 8 in each group)

**LVSP (mmHg)**	**LVEDP (mmHg)**	**LVDP (mmHg)**	**- dp/dt max (mmHg/s)**	**+ dp/dt max (mmHg/s)**	**MAP (mmHg)**	**DBP (mmHg)**	**SBP** **(mmHg)**	** Variable**
								**Group**
115.5 ± 4.1	4.75 ± 1.3	110.7 ± 3.8	2987.4 ± 234.2	4495.4 ± 301.7	85.1 ± 4.1	77.9 ± 3.8	100.4 ± 4.77	Sham
115.5 ± 9.1	4.00 ± 0.71	111.5 ± 8.7	3108.0 ± 390.8	4336.3 ± 438.3	78.6 ± 3.2	70.1 ± 3.6	95.8 ± 2.6	TBI

SBP and DBP, systolic and diastolic blood pressure, respectively; MAP, mean arterial pressure; + and - dp/dt max, maximum left ventricular contraction and relaxation velocities; LVEDP, left ventricular end diastolic pressure; LVDP, left ventricular developed pressure; LVSP, left ventricular systolic pressures. No differences (*P*>0.05) were found between TBI and sham groups in either of variables.

**Table 2:** Cardiac troponin I (cTNI) and interleukins level (mean ± SE) in heart tissue of sham and TBI groups (n = 8 in each group)

** Variable**	**IL-1 Beta** **(pg/mg tissue)**	**IL-6** **(pg/mg tissue)**	**TNF-** **(pg/mg tissue)**	**cTNI** **(ng/mg tissue)**
**Group**				
Sham	31.8 ± 5.20	13.8 ± 1.8	8.80 ± 1.6	0.260 ± 0.008
TBI	30.6 ± 4.26	12.3 ± 1.7	8.77 ± 1.4	0.259 ± 0.007

No differences (*P*>0.05) were found between TBI and sham groups in either of variables.

***Biochemical variables. ***The results for troponin-I as well as different interleukins in heart tissue and lung tissue are shown in Tables 2 and 3 and for the blood serum in Table 4. None of the measured variables showed significant differences between these groups.

**Fig. 1:**
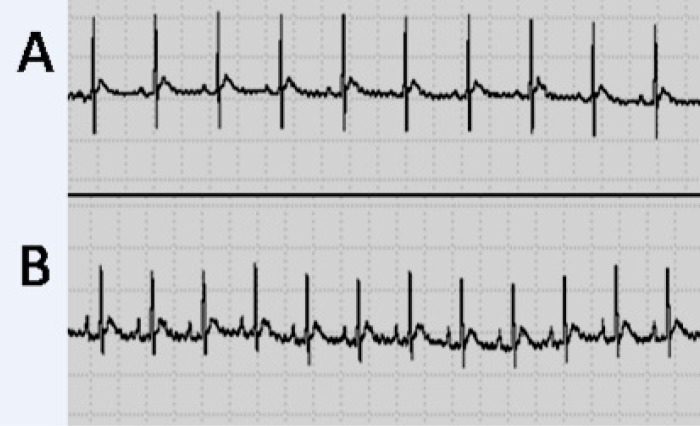
Heart and lung histopathology of study groups. A sample microscopic slide of one animal of sham group heart **(a)**, TBI group heart **(b)**, sham group lung **(c)**, and TBI group lung **(d)**. There is no sign of abnormality in heart and lung in either of animals after 24 hours of head injury. hematoxylin and eosin staining (magnification 400×).

***Histopathology. ***A sample of heart and lung pathologic assessment of test and sham groups is shown in Figure 1. Except for a little vascular congestion in heart tissue (without tissue injury) and hyperaeration in lung of TBI group, there were no important pathologic findings 24 hours after TBI. The total pathologic score for both groups was 0.0 ± 0.0 and no signs of abnormality in heart or lung sections were seen in either of the animals.

**Fig. 2:**
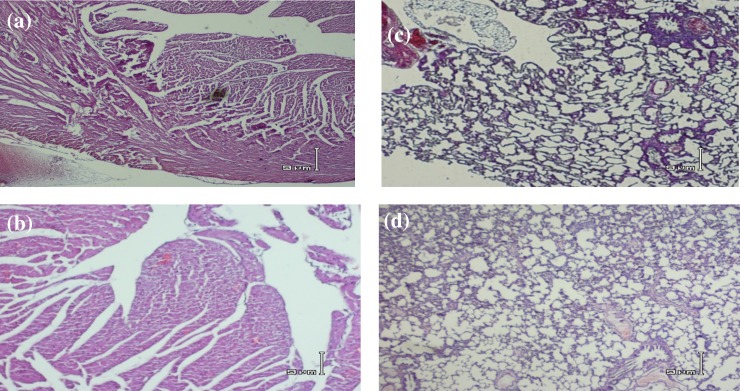
Electrical activity of the heart in study groups. A sample electrocardiography electrocardiogram (lead II) of one animal of sham **(A)** and TBI groups **(B)**. Electrocardiograph speed 50 mm/s and sensitivity 0.1 mV/mm (each large division on the recording is equivalent to 0.1 seconds on horizontal axis and 0.2 mV on vertical axis). Longer P wave duration and P-R interval in TBI group are seen. An increase in heart rate (shorter R-R interval) is seen after TBI, but overall heart rate was not significantly different between the two groups (see Table 5).

***Heart electrical activity. ***In Figure 2, a sample ECG of TBI and sham groups, and in Table 5, the results of ECG indices in both groups 24 hours after TBI are shown. Statistical comparison showed no significant difference between the indices in the two groups, except for P wave duration and P-R interval, which were significantly longer (*P*<0.05) in the TBI group.

## DISCUSSION

The main results of the present study revealed that despite cardiovascular complications such as myocardial injury that have been reported in previous studies after SAH [4, 23], there was no sign of abnormality in functional indices (Table 1), biochemical indices in heart and lung tissue (Tables 2 and 3) and in serum (Table 4), or histological indices (Fig. 1) 24 hours after diffuse TBI. It has been shown that SAH causes an increase in average blood pressure, heart rate, and cTNI [3]; however, none of which was observed in the present study. The reason for this difference in results is probably the difference between the two types of traumas, and it seems that the injury caused in diffuse TBI is basically different from that caused in SAH. The injury caused in TBI in the present study was limited to the brain and did not affect heart and lung tissues. As the studied indices were assessed after 24 hours, there was the possibility that early TBI-induced changes such as heart rate [3] and respiratory disturbances [17], and neurogenic pulmonary edema had taken place immediately and temporarily, and they had returned to normal state after 24 hours. In this regard, in our previous study of the role of estrogen and progesterone in modulating cytokine concentration following TBI, it was shown that the level of most cytokines that were increased six hours after TBI returned to about normal after 24 hours [19]. Also, veterinary coma scale that had decreased significantly in 4 hours returned to about normal value during 24 hours [17]. In the SAH model, it was also reported that post-trauma changes including excitation of sympathetic nervous system that appear in an accute phase will be decreased in the course of 60 minutes [24]. The study of Lin *et al.* [3] showed that in SAH, changes in intracranial pressure, *heart rate,* and *mean arterial pressure* are mainly immediate [3]. In another study carried out on humans, it has been reported that cardiac output is decreased at first but then increased [25]. Also, *central venous pressure* shows a decrease in the first eight hours, but then recovers. In the present study, there was no access to electron microscope; therefore, we could not rule out microstructure changes based on the lack of pathologic changes seen using an ordinary microscope. In a pathologic study on rat, brain trauma caused ultrastructural changes in heart myocytes within eight hours [4]. However, the pathologic assessment with an ordinary microscope (Fig. 1) revealed no signs of tissue damage. It is unlikely that serious damage has been happened at an ultrastructural level. On the other hand, non-significant changes in cTNI and inflammatory cytokins in both heart tissue and serum (Tables 2 and 4) support histologic findings of non-significant changes in the heart and lung tissues (Fig. 1).

**Table 3:** Interleukins level (mean ± SE) in lung tissue of sham and TBI groups (n = 8 in each group)

** Variable**	**IL-1 Beta** **(pg/mg tissue)**	**IL-6** **(pg/mg tissue)**	**TNF-** **(pg/mg tissue)**
**Group**			
Sham	160.6 ± 29.3	189.5 ± 11.7	40.7 ± 7.3
TBI	130.5 ± 23.6	210.6 ± 42.4	37.4 ± 21.6

No differences (*P*>0.05) were found between TBI and sham groups in either of variables.

**Table 4:** Cardiac troponin I (cTNI) and interleukins level (mean ± SE) in serum of sham and TBI groups (n = 8 in each group)

** Variable**	**IL-1 Beta** **(pg/ml)**	**IL-6** **(pg/ml)**	**TNF-** **(pg/ml)**	**cTNI** **(ng/ ml)**
**Group**				
Sham	12.9 ± 0.9	3.22 ± 0.16	1.31 ± 0.22	0.247 ± 0.007
TBI	13.6 ± 1.3	3.19 ± 0.22	1.32 ± 0.11	0.249 ± 0.01

No differences (*P*>0.05) were found between TBI and sham groups in either of variables.

Concerning rhythmic disorders, only signs of slow impulse conduction in atrial tissue (increase in p wave duration and P-R interval, Table 5) were observed. Van den Bergh and colleagues [8] in their study on patients with SAH reported that QT interval (which is a ventricular impulse conduction velocity index) is much longer. Slow codnuction in atrium along with no change in ventricular conduction velocity of the TBI group in the present study in comparison with normal P-R interval and long QT interval of SAH model [8] confirms that these two types of brain truma are different in mechanisms of action. It seems that SAH-induced cardiac damage is due to severe sympathetic stimulation (cathecolamine release) [3, 10] along with parasympathetic desfunction of the heart [11]. 

**Table 5:** ECG indices (means) of sham and TBI groups 24 hours after TBI (n = 8 in each group)

** Group**	**Sham**	**TBI**	***P *** **value**
**Variable**			
Heart rate (BPM)	265.5 ± 15.8	247.7 ± 15.3	0.43
R-R interval (ms)	231.9 ± 13.9	248.8 ± 14.2	0.43
P-R interval (ms)	45.0 ± 2.4	52.7 ± 2.2*	0.03
P duration (ms)	11.2 ± 1.0	15.7 ± 1.7*	0.04
QRS interval (ms)	17.2 ± 0.3	18.5 ± 0.8	0.50
QT interval (ms)	48.3 ± 0.8	54.2 ± 4.3	0.20
QTc interval (ms)	101.4 ± 3.6	109.7 ± 8.8	0.40
T peak-Tend (ms)	13.3 ± 1.5	19.8 ± 3.6	0.12
P amplitude (mV)	0.04 ± 0.02	0.08 ± 0.01	0.07
Q amplitude (mV)	0.02 ± 0.01	0.02 ± 0.01	0.46
R amplitude (mV)	0.84 ± 0.1	0.80 ± 0.1	0.60
S amplitude (mV)	-0.31 ± 0.05	-0.29 ± 0.1	0.6
ST height (mV)	0.07 ± 0.01	0.07 ± 0.02	0.68
T amplitude (mV)	0.13 ± 0.01	0.12 ± 0.03	0.60

* *P*<0.05 between TBI and Sham group. Unpaired t-test. BPM, beat per minute

Previous studies did not use diffuse TBI model or did not assess heart muscle functional indices directly, but have used indirect indices of cardiac assessment such as ecocardiogrphy or blood biochemical factors [3, 4, 9]. In the present study, in the diffuse severe brain injury (TBI) model, which is a suitable model for clinical cases, direct functional variables, such as left ventricle systolic and diastolic pressure and its contraction and relaxation velocity (+ and - dp/dt max) were assessed for the first time along with inflammatory indices, heart histopathology, electrical, and biochemical indices. In addition,assessing inflammatory and histological indices of lungs in addition to the heart made a more sound judgement on the effects of brain contusions in the first 24 hours possible. Nevertheless, due to one time-point assessments, we need to extend the evaluation to the first few hours (early responses) and the second 24 hours (more late responces) post TBI in future studies to better clarify the time-related changes in heart and lung structure and function in this model of TBI. In this regard, the measurement of the pulmonary artery pressure or oxygenation of the blood, which reaches or leaves the heart after TBI, would be more appropriate to bringing the results close to clinical conditions.

One of the limitations of the present study is that the blow was only to the head (e.g. single trauma). In reality, most of traumas due to accidents are multi-trauma consisting of both heads and chests. Therefore, the results of this study may be limited to those traumas pure to the head.

Overall, the results of assessing heart functional, histopathologic, electrical, biochemical, and inflammatory indices, and also inflammatory and histopathologic indices of the lungs were in consistent. These results show that in pure head injury in rat, after 24 hours of brain contusion, no extensive disorders appear in the two major organs of the body, namely the heart and lungs. If in clinical studies, the same results are reproduced in patients, it would enable physicians to prioritize the treatment of the damage to the brain, rendering extensive assessment of heart and lung function in the first 24 hours. Therefore, it would reduce the treatment costs for both patients and the health system.
